# Neonatal Lesser Omental Hernia Associated with Intestinal Malrotation and Congenital Duodenal Stenosis

**DOI:** 10.70352/scrj.cr.25-0787

**Published:** 2026-02-25

**Authors:** Naruki Higashidate, Kimio Asagiri, Takahiro Asakawa, Motomu Yoshida, Shiori Tsuruhisa, Kohei Yamashita

**Affiliations:** Department of Pediatric Surgery, St. Mary’s Hospital, Kurume, Fukuoka, Japan

**Keywords:** lesser omental hernia, intestinal malrotation, congenital duodenal stenosis, neonate

## Abstract

**INTRODUCTION:**

Lesser omental hernia is a rare type of internal hernia. Almost all previously reported cases are adult patients and neonatal cases are extremely rare.

**CASE PRESENTATION:**

A 2-day old female baby was referred to our hospital due to vomiting with dark colored vomitus. The abdomen was slightly distended and enteral feeding failed to establish. A gastrointestinal series revealed intestinal malrotation, and the patient was taken to the operation room. Laparoscopic investigation revealed non-rotation type intestinal malrotation without volvulus. After extraction of the entire gastrointestinal tract via umbilical incision, the liver flexure part of the transverse colon was revealed to protrude to the inferior aspect of the liver through the dorsal area of the stomach, and a diagnosis of lesser omental hernia was made. The protruded colon was reverted to its normal position and the orifices were closed by suture. Additionally, duodenal stenosis with annular pancreas was found, which were corrected by diamond-shaped duodeno-duodenal anastomosis. A gastrostomy was made before the abdomen was closed. The perioperative course was uneventful and the baby was transferred to another hospital for congenital heart disease care.

**CONCLUSIONS:**

Lesser omental hernias rarely occur congenitally. Careful exploration is necessary for neonatal gastrointestinal surgery, because rare abnormalities could occur. A 5-mm extension of the umbilical wound enabled exploration of the total alimentary tract without compromising cosmetic outcome.

## INTRODUCTION

Lesser omental hernia is a rare type of internal hernia with an incidence of 1%–4% of all internal hernias. They are reported to be most likely to occur subsequently after laparotomy such as total proctocolectomy, total colectomy, or Roux-en-Y reconstruction, and the origin of the herniation was a defect created on the omentum or gastrocolic ligament by previous surgery.^1,2)^ Although they rarely occur in patients who have not undergone abdominal surgery, the rate of herniation with congenital defect on the omentum is unclear due to its rarity. Herein, we report a neonatal case of lesser omental hernia which was found incidentally during laparoscopic surgery for intestinal malrotation. Additionally, congenital duodenal stenosis with annular pancreas was also associated.

## CASE PRESENTATION

**Fig. 1:**
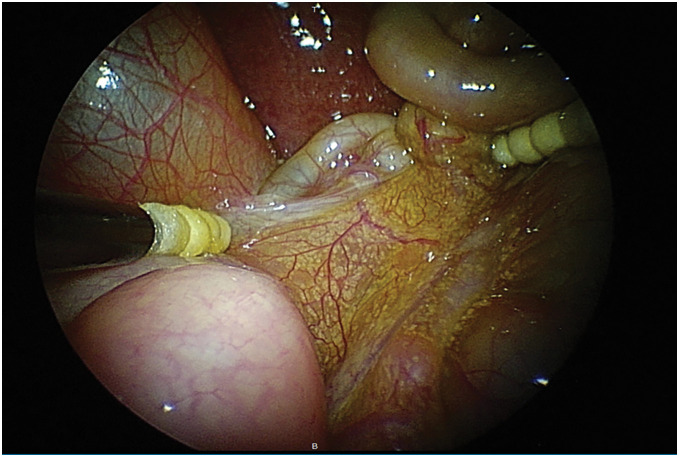
Laparoscopic investigation revealed non-rotation type intestinal malrotation without volvulus and adhesion between gallbladder and intestinal tract.

**Fig. 2:**
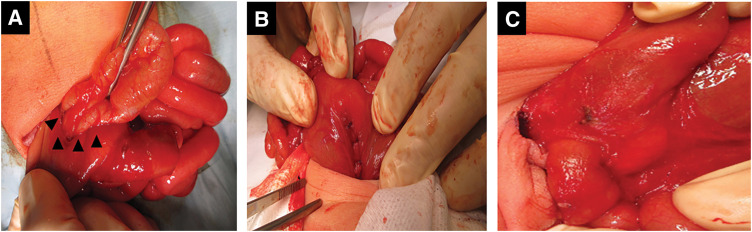
Intraoperative findings. (**A**) Ileocecum and liver flexure part of the transverse colon protruded to the inferior aspect of the liver through the dorsal area of the stomach (arrowheads). (**B**) The orifices on the lesser and greater omentum were closed. (**C**) Duodenal stenosis with annular pancreas.

**Fig. 3:**
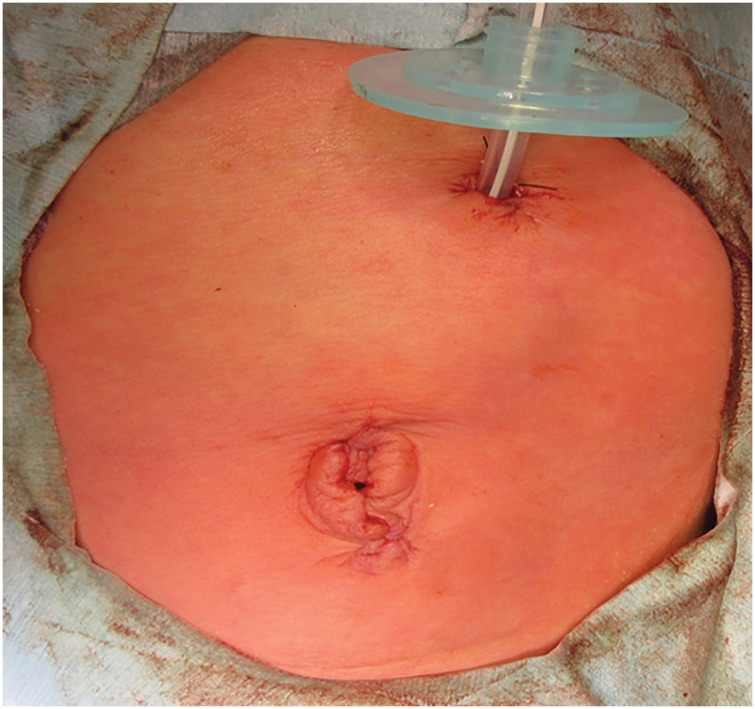
A 12-Fr. gastrostomy tube and the postoperative findings of the umbilical wound.

A 2-day old female baby was referred to our hospital due to vomiting and lethargy. She was born at 38 weeks and 0 days, with a body weight of 3261 g. The prenatal course was uneventful. Her face showed an upward slant of palpebral fissures, epicanthal folds, a small nose with a depressed nasal bridge, and macroglossia. Extremity movement was dull and muscle tone was low. Although there were no medical problems found during the prenatal period, trisomy 21 was suspected from these findings. The abdomen was slightly distended and there were no masses. The vomitus was dark brown, but contained no bilious juice. An echocardiogram showed a ventricular septum defect, patent ductus arteriosus, and double outlet right ventricle. Although milk feeding via nasogastric tube was initiated, vomiting continued and the amount of milk could not be increased sufficiently. An upper gastrointestinal series on the fifth day of birth showed that the duodenal–jejunal junction failed to cross the midline of the spine and the duodenum descended on the right side of the body. Although the outlet of contrast agent from the duodenum was slow, the flow on the distal intestine was smooth and the corkscrew sign was negative. On a lower gastrointestinal series, the ileocecal junction and appendix were located in the right upper quadrant. Therefore, a diagnosis of intestinal malrotation without midgut volvulus was made. An abdominal ultrasonography test revealed SMV rotation without midgut volvulus. We were concerned that enteral feeding deficiency was having a negative impact on the baby’s care, especially congenital heart disease which would require surgical repair. The timing of surgery was considered to be appropriate before the increase in the pulmonary artery blood flow led to progression of heart failure. Additionally, the ejection fraction was 69% on the day, therefore we concluded that the baby could tolerate laparoscopic surgery. She was taken to the operating room on the seventh day of birth after obtaining parental consent. A vertical incision was made on the umbilicus and a laparoscope was inserted. The laparoscopic investigation revealed non-rotation type intestinal malrotation without volvulus and blood supply deficiency. Adhesion between the gallbladder and intestinal tract was found and identification of anatomy was difficult due to a distended intestine (**Fig. 1**). Therefore, we enlarged the umbilical incision for 5 mm caudally and the gastrointestinal tract was extracted through the wound. After extraction, intestinal exploration revealed that the ileocecum and liver flexure part of the transverse colon protruded to the inferior aspect of the liver (**Fig. 2A**). The affected ileocecum and colon entered through the defect on the greater omentum from the ventral aspect of the bursa omentalis to the bursa omentalis; they passed through the dorsal area of the stomach, and protruded to the inferior aspect of the liver through the defect on the lesser omentum. The colon was not incarcerated and could be extracted smoothly. The orifice was observed on the lesser omentum, and a diagnosis of lesser omental hernia was made. The orifice was closed with interrupted suture using 5-0 Vicryl (**Fig. 2B**). After closure of the omental orifice, the colon was inspected, and diagnosis of non-rotation type intestinal malrotation was made due to the absence of Ladd’s ligament. Regardless of the absence of Ladd’s ligament, a narrow section was found on the descending part of the duodenum. Subsequently, we attempted Kocher’s maneuver, however, this part could not be mobilized sufficiently. A close observation revealed annular pancreas and duodenal stenosis (**Fig. 2C**). Therefore, we decided to perform a diamond-shaped duodeno-duodenal anastomosis. A 1-cm incision was made on the proximal and distal duodenal walls, and the orifices were anastomosed with interrupted suture using 5-0 Vicryl. After a 12-Fr. gastrostomy tube was placed on the stomach to secure an enteral feeding route, the abdomen was closed (**Fig. 3**). The perioperative course was uneventful and enteral feeding via the gastrostomy was initiated on the 8th POD, and there was no trouble with the enteral feeding. The baby was transferred to another hospital for treatment of congenital heart disease on POD 10.

## DISCUSSION

Lesser omental hernia is a rare type of internal hernia with an incidence of 1%–4% of all internal hernias.^1)^ There were no previous reports of a neonatal case of lesser omental hernia as far as we could find. To date, lesser omental hernias have been reported to occur subsequently after laparotomy such as total proctocolectomy, total colectomy, or Roux-en-Y reconstruction in most cases, and the origin of the herniation was a defect created on the omentum or gastrocolic ligament by previous surgery.^2)^ On the other hand, it rarely occurs in patients who have not undergone abdominal surgery, and a congenital defect on the omentum is considered to be involved in herniation.^3)^ The omentum and bursa omentalis develop in the 4–8th prenatal week from the ventral and dorsal mesogastrium. Gastric primordia rotation results in stretching of the ventral mesogastrium to the right-cranial direction and the dorsal mesogastrium to the left-caudal direction. The extended ventral mesogastrium connects to the inferior aspect of the liver and forms the lesser omentum, and the extended dorsal mesogastrium forms the bursa omentalis and the inferior part of it conglutinates and forms the greater omentum. Omental hernia occurs due to failure in agglutination during the process.

Previously, Kitagishi advocated two types of classification of lesser omental hernia; Type 1: protrusion via the lesser omental orifice into the lesser sac, and Type 2: protrusion via the foramen of Winslow or a retrogastric space created by colectomy into the abdominal cavity.^4)^ In the present case, the route of herniation of the colon was from the greater omental orifice through the bursa omentalis to the lesser omental orifice. Thus, the present case could not be applied to the above two types. In addition, another classification was proposed by Chen et al.^5)^ According to the classification, Type 1: herniation through the greater omentum, Type 2: herniation through the foramen of Winslow, Type 3: herniation through the transverse mesocolon, and Type 4: direct herniation through the lesser omentum to the bursa omentalis. The present case would belong to type 1 among Chen’s classification.

Alves reviewed 30 cases of previously reported lesser omental hernias, and the age of the patients was distributed from 14 to 88 years.^6)^ The affected alimentary tract was the small intestine in 26 cases (87%), the colon in 3 cases (10%) and the duodenum and uncinated part of the pancreas in 1 case (3%). Additionally, associated hernia was seen in 18 cases (60%), mesocolon in 4, gastrocolic ligament in 11, and Winslow in 3 cases. Colon herniation is relatively rare in lesser omental hernia as mentioned above. In the present case, although the intestinal malrotation and lesser omental hernia were independent congenital anomalies that coincidentally occurred together, the lack of fixation of the ascending and liver flexure part of the colon due to intestinal malrotation was involved in herniation of the colon instead of the small intestine. Additionally, another review of 15 cases by Konishi et al. revealed that bowel resection was necessary due to necrosis in 5 cases and the diameter of the orifice ranged from 1.5 to 5 cm.^7)^ Although the affected colon was not incarcerated and colon obstruction was not observed because of the relatively large orifice in the present case, we closed the orifices on both the lesser and greater omentum to prevent recurrent protrusion.

Recently, the advantages of the laparoscopic approach for neonatal intestinal malrotation were advocated. In 2024, Johnston et al. reported that laparoscopic surgery was superior to open surgery in terms of low volume of blood loss, less time to achieve regular diet, less time to evacuate first stool, and low incidence of adhesion formation.^8)^ However, in patients with midgut volvulus, some authors are concerned about the higher rate of recurrence of volvulus after laparoscopic surgery.^9–11)^ Additionally, Hsiao et al. advocated that the laparoscopic approach for intestinal malrotation is an alternative method and should be limited to patients in a stable condition and without volvulus.^12)^ In the present case, the diagnosis of intestinal malrotation without midgut volvulus was made by preoperative imaging studies and the laparoscopic approach was indicated. However, prolongation of the umbilical incision for 5 mm was required for identification of anatomy. It enabled exploration of the total alimentary tract from the stomach to the rectum, and we could identify duodenal stenosis with annular pancreas and a rare anomaly of lesser omental hernia. A vertical incision on the umbilicus for insertion of the laparoscope, and trivial extension of the wound yielded sufficient surgical field without compromising on the cosmetic outcome.

## CONCLUSIONS

Lesser omental hernias rarely occur congenitally. In the present case, the colon was involved due to the lack of its fixation resulting from intestinal malrotation. Careful exploration is necessary for neonatal gastrointestinal surgery, because rare anomalies could be associated. A 5-mm extension of the umbilical wound enabled exploration of the total alimentary tract without compromising the cosmetic outcome.

## DECLARATIONS

### Funding

This research did not receive any specific grant from funding agencies in the public, commercial, or not-for-profit sectors.

### Authors’ contributions

NH acquired the data and drafted the manuscript.

KA coordinated the study and helped draft the manuscript.

All authors have read and approved the final manuscript.

### Availability of data and materials

The datasets supporting the conclusions of this article are included within the article.

### Ethics approval and consent to participate

This work does not require ethical considerations or approval. Informed consent to participate in this study was obtained from the patient’s parents.

### Consent for publication

Written informed consent was obtained from the patient’s parents to publish this article.

### Competing interests

We have no competing interests.
